# High-Sensitivity Large-Area Photodiode Read-Out Using a Divide-and-Conquer Technique

**DOI:** 10.3390/s20216316

**Published:** 2020-11-05

**Authors:** Guillermo Royo, Carlos Sánchez-Azqueta, Concepción Aldea, Santiago Celma

**Affiliations:** 1 Department of Electronic Engineering and Communications, Universidad de Zaragoza, 50009 Zaragoza, Spain; caldea@unizar.es (C.A.); scelma@unizar.es (S.C.); 2 Department of Applied Physics, Universidad de Zaragoza, 50009 Zaragoza, Spain; csanaz@unizar.es

**Keywords:** integrated photodiode, low-noise amplifier, noise reduction techniques, photodiode read-out, transimpedance amplifier

## Abstract

In this letter, we present a novel technique to increase the sensitivity of optical read-out with large integrated photodiodes (PD). It consists of manufacturing the PD in several pieces, instead of a single device, and connecting a dedicated transimpedance amplifier (TIA) to each of these pieces. The output signals of the TIAs are combined, achieving a higher signal-to-noise ratio than with the traditional approach. This work shows a remarkable improvement in the sensitivity and transimpedance without the need for additional modifications or compensation techniques. As a result, an increase in sensitivity of 7.9 dBm and transimpedance of 8.7 dBΩ for the same bandwidth is achieved when dividing the photodiode read-out into 16 parallel paths. The proposed divide-and-conquer technique can be applied to any TIA design, and it is also independent of the core amplifier structure and fabrication process, which means it is compatible with every technology allowing the integration of PDs.

## 1. Introduction

Large area photodiodes (1-mm diameter or more) are used in many instrumentation and communication applications. For example, plastic optical fiber (POF) sensors are a new class of fiber sensors used in oil, gas, biotechnology, and energy fields. Thanks to the POF’s large diameter and the inexpensive peripheral components and low installation costs, many electronic pieces of instrumentation are built based on POF and photodiodes with a large active area, such as a sensor for oil trucker valve monitoring, a monitoring system for high voltage substation switch, an oil leaking sensor for offshore platforms, and a solar tracker for illumination [1]

On the other hand, transmitting data at a high transmission rate is crucial in today’s world. We are constantly connected and demanding more and more information. The demand is continuously growing, and communication networks must be prepared for this evolving scenario. This need has driven the use and development of optical communication systems over the last years due to their capability to transmit information at very high data rates. However, because of the high cost of installation and maintenance of glass optical fiber (GOF) systems, the vast majority of the currently deployed short-range networks are still based on copper, which cannot transmit information as fast as optical fibers.

In this context, a promising solution to overcome the short-range bottleneck is plastic optical fibers (POF), which can provide a higher data rate and are more robust than copper cables, with the additional advantage of their immunity to electromagnetic interferences. They have been recently used in the automotive industry since they are lighter and more flexible than copper cables and also show a few advantages over GOF, such as higher stability against vibrations thanks to their much larger core diameter [2]. They are also easier to install and manipulate, thus reducing the costs of installation and maintenance, making POF cost-competitive and an excellent candidate for short-distance applications, such as home networks.

On the contrary, POF suffers from high attenuation (0.2 dB/m at 650 nm), and coupling with both the transmitter and the receiver generates even higher losses. Over the last few years, great efforts have been made to increase the range and throughput of these systems by improving the performance of the photonic devices and the electronic sensor interfaces [2,3,4,5]. In addition, since the core of a step index POF (SI-POF) has a 1-mm diameter, large-area optical sensors are required to achieve high-efficiency light coupling [6]. It is well known that large area photodiodes (PD) have an important parasitic capacitance of the order of several picofarads (pF) [7]. Therefore, to interface these photonic devices and overcome the high attenuation of POF, a low-noise transimpedance amplifier (TIA) with low input impedance must be designed.

**Figure 1:**
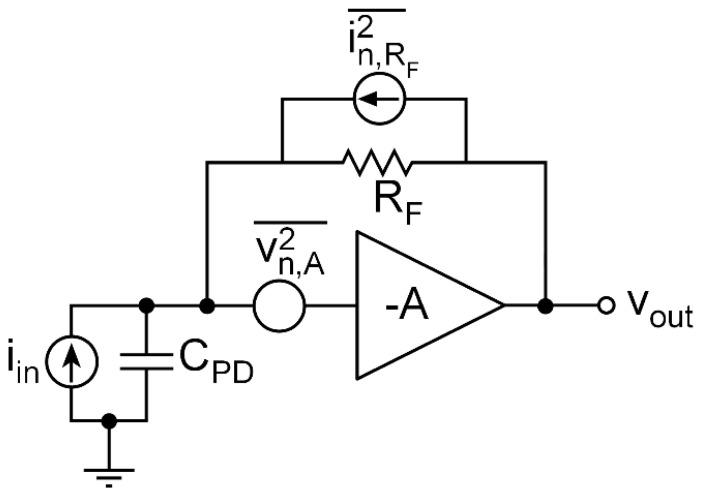
Schematic diagram of a shunt-feedback transimpedance amplifier (TIA), including noise sources.

To increase the transmission length, highly sensitive optical receivers with large area photodiodes with a diameter of the order of 1-mm must be used. Several TIAs have already been proposed for POF applications, the shunt-feedback topology, shown in Figure 1, being the most commonly employed due to its more linear performance and ease of design. Moreover, there are several noise reduction techniques described in the literature to increase receiver sensitivity [8,9]. However, it is still very challenging to fulfill the requirements of sensitivity and bandwidth (BW) needed for the low-cost applications mentioned above simultaneously due to the high capacitance of the photodiodes and their small responsivity. Improving these characteristics is critical to allow the possibility of reaching longer distances and achieving higher data transmission rates.

In this letter, we present a novel technique to increase the sensitivity of optical read-outs based on a feedback TIA with a large integrated photodiode since this topology is the most commonly employed in photodiode read-out designs [10]. Nevertheless, the proposed technique can be applied independently of the TIA core amplifier topology, and, thus, it can be used in any silicon photonics technology. In Section 1, a background is given to introduce the context and provide motivation for this work. Section 2 describes the proposed noise reduction technique with a theoretical analysis, and in Section 3, the technique is applied to a transistor-level simulation of a shunt-feedback TIA to show post-layout results of this study. Finally, Section 4 summarizes the conclusions of this work.

## 2. Technique Description

**Figure 2:**
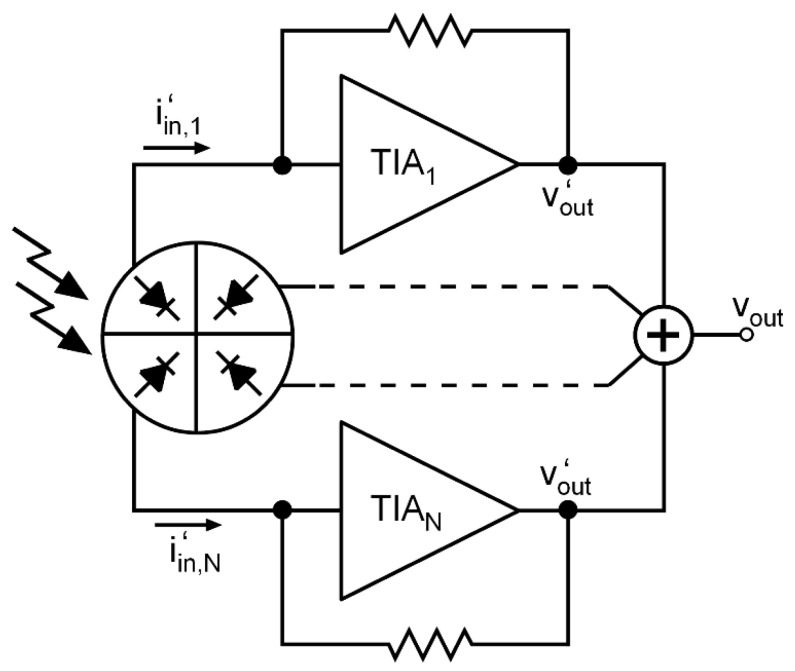
Conceptual scheme of a photodiode read-out using the sliced- photodiode (PD) technique with N TIAs.

One of the main challenges of the TIA design is to overcome the large parasitic capacitance of the PD to achieve wide bandwidth and low noise. In the technique described in this letter, we propose slicing the photodiode and manufacturing it in N individual pieces, connecting a TIA to each one of them, as shown in Figure 2, with the aim of reducing the equivalent input-referred noise. Slicing the PD helps reduce the difficulty of designing the TIA since the parasitic capacitance of each PD piece is smaller, and, thus, the TIA design constraints are relaxed.

To understand the operating principle of this technique, we must first recall the approximate modulus of the complex transimpedance, $Z_{T}$, and the input-referred noise, $\bar{i^{2}{}_{n,IN}}$, of a shunt-feedback TIA as a function of the angular frequency, ω:

(1) $$\left|Z_{T}\right|=\left|\frac{R_{F}}{1+j\frac{R_{F}C_{in}}{A}\omega }\right|$$

(2) $$\bar{i^{2}{}_{n,\;IN}}\approx \frac{K^{2}}{\omega R_{F}^{2}}+\frac{4K_{B}T}{R_{F}}+\frac{\bar{v_{n,A}^{2}}}{R_{F}^{2}}+\bar{v_{n,A}^{2}}\omega^{2}C_{in}^{2}$$

where K is a constant relative to the 1/f-dependent Flicker noise contribution, $K_{B}$, is the Boltzmann’s constant, *T* the absolute temperature, $R_{F}$ the feedback resistor, A and $\bar{v_{n,A}^{2}}$ the open-loop gain and input voltage noise of the core amplifier, respectively, and $C_{in}$ the equivalent input capacitance, which includes the PD capacitance, $C_{PD}$ and the input capacitance of the core amplifier.

It is well known that the tradeoff between bandwidth, transimpedance, input capacitance, and the technological transition frequency limitation, $f_{T}$, leads to the transimpedance limit described in [11]:

(3) $$R_{T}\leq \frac{A_{0}\omega_{A}}{C_{in}BW^{2}}$$

where $R_{T}$ is the transimpedance at zero frequency and $A_{0}\omega_{A}$ is the gain-bandwidth product of the voltage amplifier, roughly proportional to the technology parameter $f_{T}$. Therefore, from (3), it is clear that, for a target BW, the maximum achievable transimpedance is bound to the capacitance at the input node. Despite the intrinsic capacitance of the PD being lower when biased at high reverse voltage, this parameter should be treated as constant. Thus, to reduce $C_{in}$. The only possible way is to make a greater effort in the TIA design to minimize its input capacitance. Moreover, according to (2), the input-referred noise is strongly dependent on the input-node capacitance, since the third term, the $f^{2}$-noise is proportional to $C_{in}^{2}$. Therefore, if a lower value of the input capacitance is achieved, not only a greater transimpedance can be reached, but also lower noise and, thus, better sensitivity. In the proposed technique, we explore the possibility of dividing the PD into N pieces to obtain a 1/N parasitic capacitance for each one. To do that, the PD should be manufactured in *N* slices, and each of them should be connected to an individual TIA, as shown in Figure 2. PDs in silicon photonics are implemented as an array of multiple fingers, which facilitates their division [12,13].

The first and immediate advantage of this technique is a much easier design of the TIA since the parasitic capacitance of the PD that it is attached to is now N times smaller so that achieving wide BW should be much simpler.

Moreover, according to (2), since the input capacitance is approximately divided by N, the $f^{2}$-noise contribution decreases by a factor of $N^{2}$. Since the signal power received by each PD piece is reduced linearly with the number of slices, the equivalent input-referred noise should decrease. Let us explore this hypothesis and compare it with the traditional approach: After slicing the PD into *N* pieces, each piece now receives an optical power of 1/N times the total optical signal power, and, therefore, it generates a current $i_{in}^{\prime }=i_{in}/N$. The capacitance of each input node is now $C_{in}^{\prime }≃C_{in}/N$, since the area of each PD piece is *N* times smaller than the total PD area and $C_{PD}$ is the major contribution to $C_{in}$. This technique works as long as $C_{PD}≃C_{in}$, that is, it can be applied to TIA designs using large integrated PDs with high intrinsic capacitance, such as the ones employed in [14] and [15], where large PD capacitances of 14 pF and 64 pF are reported, respectively.

Regarding the topology, design parameters and transistor sizing of the core amplifier, we can use the same values to compare both approaches. However, to keep a constant BW and quality factor, each TIA must keep a constant $R_{F}C_{in}$ product, thus a feedback resistor $R_{F}^{\prime }=N\cdot R_{F}$ will be used on the sliced-PD with multiple-TIA design [16]. Therefore, after slicing the PD into N equal pieces, each TIA achieves the same bandwidth as before and generates the same output voltage:

(4) $$v_{out}^{\prime }=i_{in}^{\prime }R_{F}^{\prime }=\frac{i_{in}}{N}\cdot NR_{F}=i_{in}R_{F}$$

and the input-referred noise of each TIA, $\bar{i^{2}{}_{n,IN}\prime }$ will be:

(5) $$\bar{i^{2}{}_{n,\;IN}\prime }\approx \frac{K^{2}}{\omega N^{2}R_{F}^{2}}+\frac{4K_{B}T}{NR_{F}}+\frac{\bar{v_{n,A}^{2}}}{N^{2}R_{F}^{2}}+\frac{\bar{v_{n,A}^{2}}\omega^{2}C_{in}^{2}}{N^{2}}$$

which shows a reduction in the input noise by a factor close to 1/N.

Since all the N signals are synchronized, we can combine the outputs to obtain a higher transimpedance, $R_{T}$, than with a single-piece PD configuration, so that:

(6) $$R_{T}=\frac{v_{out}}{i_{in}}=\overset{N}{\underset{1}{\sum }}i_{in}R_{F}=NR_{F}$$

Moreover, since each path from each PD slice is independent of each other, the electrical noise of each TIA is uncorrelated. Therefore, we can calculate the total input noise with a quadratic sum of the N noise contributions as:

(7) $$\begin{array}{ll} \bar{i^{2}{}_{n,IN}} & =\overset{N}{\underset{1}{\sum }}\frac{K^{2}}{\omega N^{2}R_{F}^{2}}+\frac{4K_{B}T}{NR_{F}}+\frac{\bar{v_{n,A}^{2}}}{N^{2}R_{F}^{2}}+\frac{\bar{v_{n,A}^{2}}\omega^{2}C_{in}^{2}}{N^{2}}\\ & =\frac{K^{2}}{\omega NR_{F}^{2}}+\frac{4K_{B}T}{R_{F}}+\frac{\bar{v_{n,A}^{2}}}{NR_{F}^{2}}+\frac{\bar{v_{n,A}^{2}}\omega^{2}C_{in}^{2}}{N} \end{array}$$

This is a remarkable result, since the $f^{2}$-noise term, which is the dominant contribution to the bandwidth-integrated noise, shows an inverse dependence with N, a better signal-to-noise ratio (SNR) can be achieved.

## 3. Results

**Figure 3:**
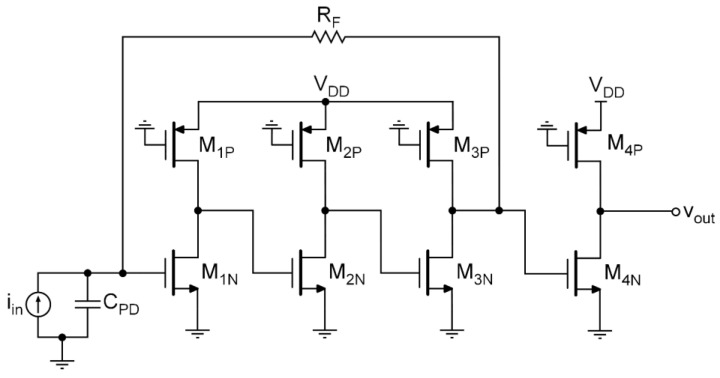
Transistor-level topology of the transimpedance amplifier employed in the simulations to test the proposed noise-reduction technique.

Let us now apply the sliced photodiode technique to an actual feedback TIA design. Since the technique can be employed with the independence of the core-amplifier design, in this work, the configuration employed to perform the simulation was a TIA consisting of three cascaded common-source stages with a negative resistive loop, as shown in Figure 3. An output common-source buffer was included to employ it as a simple signal adder. The circuit was implemented in 65-nm CMOS technology with a single 1.2-V voltage supply. To model the PD, we used the PD parameters reported in [14], that is $C_{PD}=14\;\mathrm{pF}$ and a responsivity of 0.42 A/W at 850 nm. As the main purpose of this letter is to demonstrate the feasibility, the proposed divide-and-conquer technique, we chose a BW of 1 GHz, for which we optimized the TIA design, achieving a maximum sensitivity of −11.0 dBm. All simulations were performed using the Cadence Spectre Simulation Platform with a BSIM3v3.2 level 53 transistor model for the TSMC 65-nm CMOS technology.

Next, as a first approximation, we divided the PD into N equal pieces and replicated the TIA N times, using a feedback resistor N times larger and the same voltage amplifier as in the original design. By combining the output signals of each TIA, we increased the SNR by a factor of roughly $\sqrt{N}$. Since the voltage signals are summed linearly, and the uncorrelated noise sum is quadratic, we ended up obtaining the equivalent input-referred noise expression (7), effectively reducing it and improving the sensitivity of the read-out.

**Figure 4:**
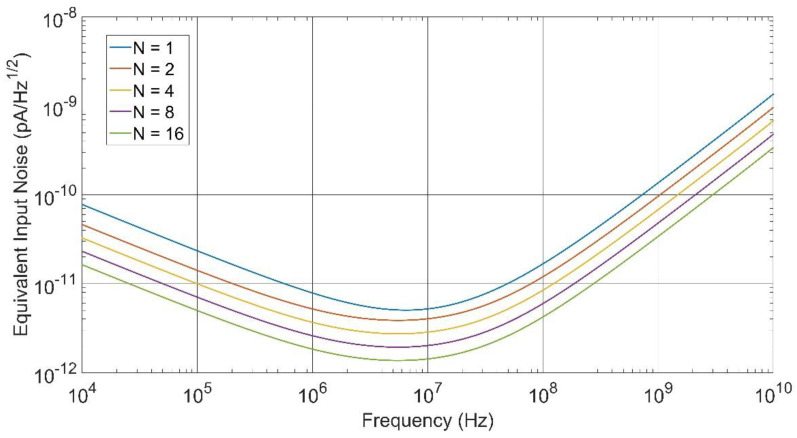
Input-referred noise of the front-end using the divide-and-conquer technique for different numbers of pieces.

In this work, the sliced PD technique was applied, dividing the PD into N pieces, choosing powers of 2 for the values of N, up to 16. To combine the output signals of each TIA, we employed the output buffer, splitting the transistor $M_{4N}$ shown in Figure 3 into N equal transistors to implement a simple signal adder. Since the transconductance of each transistor was now divided by N, the output signal should remain similar to the single-PD case, but the quadratic sum of the noise contributions should provide an increase in the SNR. Figure 4 shows the equivalent input-referred noise response, clearly exhibiting a greater decrease in the spectral density at high-frequencies for higher N values. It is clear that a decrease in the $f^{2}$-noise contribution was achieved, leading to a better SNR and, thus, higher sensitivity. To calculate the sensitivity of the TIA, we considered a 1.25 Gb/s pseudo-random bit sequence (PRBS) non-return-zero binary data transmission with a bit error ratio (BER) better than $10^{-12}$.

Table 1 summarizes the key performance parameters of the front-end obtained by slicing the PD into different numbers of pieces. There was a remarkable increase in the sensitivity, measured for a BER of $10^{-12}$, from −11.0 dBm using the traditional single-PD approach to −15.8 dBm by slicing the PD into 16 pieces. This means that using this technique, we obtained an improvement of 4.2 dBm. Notice that the noise was three times lower even though the bandwidth increased by 32%. The main drawback is power consumption, which increases by a factor of N.

As mentioned above, the TIA design was bonded to the intrinsic capacitance of the PD. After slicing the PD, the capacitance was lowered by a factor of N. Therefore, in a second case, we optimized the TIA with N=16 to compare the performance of an optimized 16-pieces sliced-PD versus the optimized single-PD approach. Table 2 summarizes the design parameters of each optimized design approach for a 1-GHz BW and compares their performances.

A much higher transimpedance was achieved after optimizing the TIA for the 16-piece sliced-PD case. Although the increase in power consumption by a factor of 13 cannot be ignored, it is remarkable that the input RMS noise was lowered by a factor of 6, improving the sensitivity by almost 8 dBm.

Finally, it is noteworthy that an optimized design with a single PD and the same power budget did not improve performance to the same extent as the proposed technique.

## 4. Conclusions

A novel photodiode read-out design technique to use with large active area photodiodes has been presented in this letter. It consisted of slicing the photodiode area and connecting a TIA to each piece, instead of the conventional single-PD approach. The simulation post-layout results showed that the sensitivity was improved while maintaining the bandwidth and also that the achieved transimpedance could be much higher when the technique was applied, dividing the PD into a large number of pieces. In this letter, we have shown that the proposed divide-and-conquer technique is a good candidate to improve the sensitivity of POF equipment with high junction capacitance integrated PDs, and it can potentially be employed in a vast amount of applications where optical sensors with large-area photodiodes are required. Moreover, it does not depend on the transistor-level architecture of the TIA, which makes it compatible with almost every TIA design.

## Figures and Tables

**Table 1:** Summary of the simulation results using the sliced-PD technique with different numbers of slices.

Parameter	*N* = 1	2	4	8	16
$R_{T}$ (dBΩ)	75.7	75.7	75.8	75.8	75.7
BW ^1^ (GHz)	1.02	1.20	1.31	1.34	1.35
Input RMS Noise (μA)	4.74	3.89	2.57	1.89	1.58
Sensitivity *	−11.0	−11.8	−13.7	−15.0	−15.8
Power ** (mW)	2.9	5.8	11.5	23	46

^1^ 3-dB bandwidth (BW); * Measured sensitivity for the transmission of a 1.25 Gb/s pseudo-random bit sequence (PRBS) with non-return to zero (NRZ) encoding with a bit error ratio (BER) of 10^−12^; ** Total electrical power consumption of the transimpedance amplifier.

**Table 2:** Comparison of the simulation results with optimized parameters for both approaches, the traditional approach, and applying the sliced-PD, respectively.

Parameter	Single PD Fixed BW	Single PD Fixed Power	16 Sliced-PD
$R_{T}$ (dBΩ)	75.7	80.3	84.4
BW (GHz)	1.02	1.21	1.02
Input RMS Noise (μA)	4.74	1.82	0.78
Sensitivity (dBm@BER = 10^−12^)	−11.0	−15.1	−18.9
Power (mW)	2.9	38.8	38.8
